# Primary lipomatous tumor/well-differentiated liposarcoma of the breast with invasion of the pectoralis major muscle: A case report

**DOI:** 10.1016/j.ijscr.2025.111680

**Published:** 2025-07-16

**Authors:** Guochun Qiu, Xiaoyu Zhou, Zhongming Shang, Yuping Wang, Rui Hai

**Affiliations:** a Department of Vascular, breast, thyroid surgery, the Affiliated Hospital of traditional Chinese medicine of Southwest Medical University, Luzhou 646000, China; b Department of gynaecology, the Affiliated Hospital of traditional Chinese medicine of Southwest Medical University, Luzhou 646000, China

**Keywords:** Breast tumor, Lipomatous tumor/well differentiated liposarcoma, Pectoralis major muscle

## Abstract

**Introduction and importance:**

Atypical Lipoma/Well Differentiated Liposarcoma (ALT/WDLS) is a moderate malignant soft tissue sarcoma originating from mesenchymal tissue. It is the most common subtype of liposarcoma, typically found in the retroperitoneum and limbs. It is extremely rare in the breast. Due to its mild morphology and polymorphic changes associated with various subtypes, ALT/WDLS can easily be mistaken for other benign breast tumors. Accurate diagnosis requires careful evaluation of different morphological features, molecular genetic changes, and clinical characteristics.

**Case introduction:**

This article reports a case of breast ALT/WDLS in a 68 year old female. Examination indicates a mass in the right chest, with unclear boundaries with adjacent chest wall muscle. Subsequently, surgical treatment was performed, and pathological results confirmed ALT/WDLS.

**Clinical discussion:**

It is rare for ALT/WDLS to originate from the breast. When dealing with breast adipose tissue masses, it is necessary to carefully distinguish the clinical manifestations of benign lipomas and ALT/WDLS. Although ALT/WDLS is moderately malignant, its imaging and clinical similarities to benign lipomas should not be taken lightly in diagnosis. Pathological evaluation and molecular biology examination of tumors can provide important diagnostic criteria for differentiation.

**Conclusion:**

This report emphasizes the need to differentiate benign lipomas from ALT/WDLS when evaluating breast adipose tissue masses. This case also highlights the importance of early detection and accurate diagnosis, reminding clinicians to remain highly vigilant when facing similar cases and to conduct comprehensive diagnosis and treatment through multidisciplinary evaluation.

## Highlights

• Extremely Rare Presentation: Primary breast ALT/WDLS with pectoralis major invasion, among <20 documented cases globally.
• Critical Diagnostic Challenges:Mimics benign lipomas; MDM2/CPM testing (100% sensitivity/specificity) and red flags (age > 50, rapid growth, deep location) are critical.
• Novel Imaging Correlations: Heterogeneous fat/soft-tissue density, 2-mm fibrous septa (spoke-wheel), ill-defined tumor-muscle interface (>50 HU/cm gradient).
• Evidence-Based Management Protocol:High-risk patients:3-month CT + molecular testing; standard: 6-month CT + annual MDM2/CDK4 for 5 years.
• Translational Research Implications:CPM as a diagnostic biomarker; molecular stratification for personalized follow-up.

## 1 Introduction

This case report follows the SCARE criteria exclusively to ensure a structured and comprehensive presentation of the clinical scenario [1]. Liposarcoma is a rare connective tissue tumor that originates from adipocytes and accounts for approximately 20 % of all soft tissue malignancies. It is the most common type of sarcoma in adults. Its subtypes include atypical lipoma/well differentiated liposarcoma (ALT/WDLS), dedifferentiated lipoma (DDLPS), mucinous liposarcoma, and pleomorphic liposarcoma [2,3]. Among these, ALT/WDLS is the most commonly encountered pathological subtype, primarily affecting the extremities and retroperitoneum [4]. Here, we report a case of breast ALT/WDLS with invasion of the pectoralis major muscle, discussing its clinical characteristics, morphological features, and molecular genetic alterations, with the aim of providing insights for clinical diagnosis and treatment.

## 2 Case presentation

**Fig. 1:**
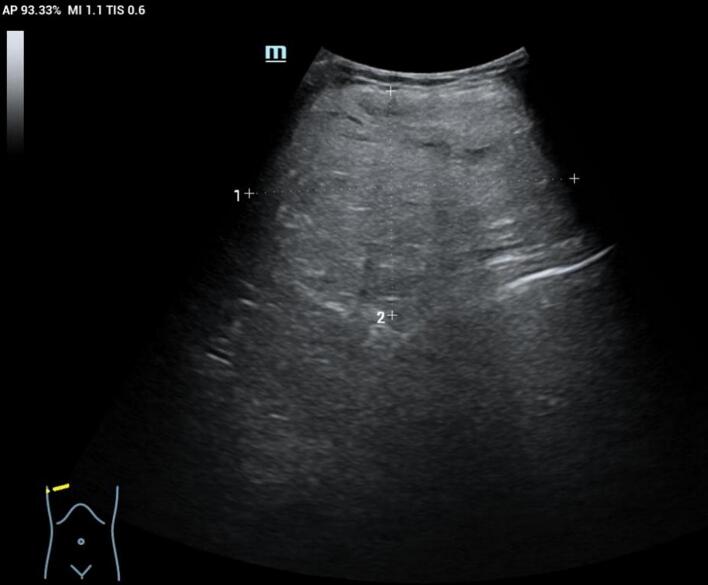
Breast ultrasound shows heterogeneous and slightly strong echoes in the upper quadrant of the right breast, with a regular shape and visible boundaries.

**Fig. 2:**
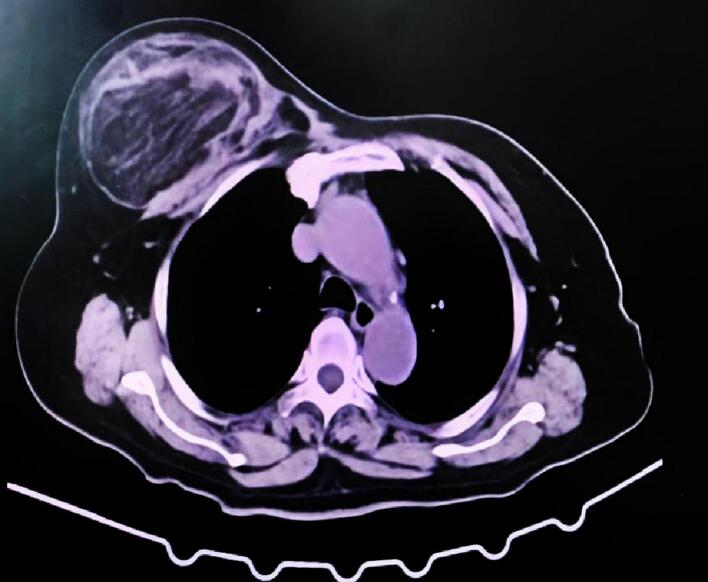
Chest CT shows a mixed density mass shadow in the right breast, with unclear demarcation from adjacent chest wall muscles.

**Fig. 3:**
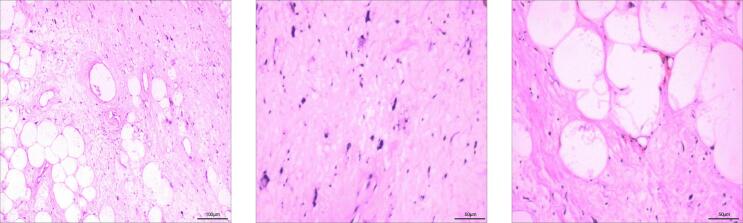
HE staining of tumor slices indicates the presence of distinct fibrous bundles within the tumor and a clear boundary between the surrounding lipoma like tissue.

A 68-year-old female patient presented with a one-year history of a rapidly growing palpable mass in her right breast, which prompted her to seek medical attention. The patient had a medical history of coronary artery disease and hypertension, both of which were well-controlled with regular medication. Physical examination revealed no systemic symptoms (including absence of weight loss or anorexia), no nipple discharge or skin changes, and no palpable axillary lymphadenopathy. The family history was negative for both breast and ovarian cancer. Clinical examination revealed a large mass, approximately 20 × 10 cm, in the upper quadrant of the right breast. The mass was non-tender, well-defined, mobile, without any skin changes or nipple discharge, and there were no palpable axillary lymph nodes. The mammogram shows a large lump of approximately 18.6 × 9 cm in the upper quadrant of the right breast. Ultrasonographic examination showed an 18.4 × 10.8 × 7.1 cm heterogeneous, slightly hyperechoic mass in the upper quadrant of the right breast, located 9.7 mm below the areola. The mass had a relatively regular shape, with well-defined boundaries, and no enlarged lymph nodes were noted in either axilla (Fig. 1). Chest CT scan revealed a mixed-density mass in the right anterior chest, measuring approximately 18 × 10 cm, with an unclear boundary with the adjacent chest wall muscles, suggestive of a potential mesenchymal tissue origin (Fig. 2). Core needle biopsy indicated a lipoma of the right breast. The patient underwent a right breast lump resection surgery. During the surgery, a mass of approximately 18 × 10 cm was found in the upper quadrant of the right breast. The lump is oval shaped, hard, soft in texture, and has a distinct outline. It is closely integrated with the pectoralis major muscle, and considering the invasion of tumor tissue into the pectoralis major muscle, further wide local excision was performed. Intraoperative frozen section results indicated a fat-originating tumor in the right breast, with scattered pleomorphic cells, the nature of which was indeterminate (Fig. 3). Postoperative pathology confirmed a diagnosis of ALT/WDLS of the right breast. The tumor measured 18 × 11 × 8 cm. Immunohistochemical staining results were as follows: Vimentin (+), S-100 (−), CD34 (−), SMA (−), P16 (+), WT-1 (−), CD68 (+), Ki-67 (+, 5 %), MDM-2 (+++), and CDK4 (++). The patient recovered well postoperatively with no signs of recurrence. Follow-up monitoring included semiannual chest and abdominal CT scans, quarterly serum LDH and alkaline phosphatase tests, and annual MDM2/CDK4 expression analysis, along with monthly self-examination training and symptom monitoring education for the patient.

## 3 Discussion

ALT/WDLS predominantly affects individuals aged 40 to 80, with a higher incidence in males compared to females. It is most commonly observed in the retroperitoneum and extremities, followed by the vicinity of the testes, inguinal region, and spermatic cord. Rarely, it may occur in the mediastinum, thoracic cavity, head and neck, and vulva [4]. The concept of ALT/WDLS was first introduced by Evans et al. [5] in 1979. Both well-differentiated liposarcoma and atypical lipoma-like tumors share identical histological, biological, and chromosomal profiles. The terminology ALT or WDLS is primarily determined by the tumor's location and whether complete surgical excision is possible. The term ALT refers to tumors located in surgically accessible areas, such as the limbs and trunk, where radical excision is feasible and curative. In contrast, WDLS refers to tumors located in anatomically challenging regions, such as the retroperitoneum and mediastinum, where complete surgical resection is not always achievable, leading to recurrent local recurrences and potentially fatal consequences [6]. The majority of ALT/WDLS cases present as slow-growing, painless masses in deep soft tissues, with few clinical symptoms. Patients typically seek medical attention only when the tumor reaches a larger size. This case reports a primary breast ALT/WDLS with pectoralis major involvement, presenting with rapid growth, a very rare clinical manifestation. To date, <20 cases have been reported in the literature.

The pathological characteristics of ALT/WDLS are generally manifested as a large, well-defined, gray-yellow, lobulated mass [7]. The tumor tissue consists primarily of mature adipose tissue, which closely resembles a lipoma in appearance. In some regions of the tumor, scattered, large, deeply stained lipoblasts and atypical adipocytes are observed, indicating a certain degree of atypia. Additionally, fibrous septa or lobular partitions, varying degrees of myxoid degeneration, and areas of adiponecrosis are present within the tumor. Calcification and ossification may also occur in some tumor tissues [8]. In this case, the tumor appeared yellow with areas of calcification, and distinct fibrous bundles were observed separating the tumor from the surrounding lipoma-like tissue. It is crucial to differentiate between breast ALT/WDLS and benign breast lipoma preoperatively, as both share similar clinical features and imaging findings, which can lead to diagnostic confusion. In the radiological differentiation between breast lipomatous tumors and ALT/WDLS, the CT characteristics of our case demonstrate several diagnostically significant features: First, the tumor presented as a large 18 cm mass with heterogeneous density (fat-density regions measuring −80 to −30 HU and soft-tissue components ranging +20 to +40 HU). These findings correlate strongly with the study by Kransdorf et al., which reported that liposarcomas averaged significantly larger diameters (23.5 cm) than lipomas (12.5 cm) (*P* < 0.001), with lesions containing <75 % fatty tissue showing an 8.3-fold increased probability of malignancy (95 % CI 2.0–33.3) [9]. Second, the presence of fibrous septa >2 mm thick displaying a characteristic “spoke-wheel” distribution pattern corresponds precisely with Kransdorf's established criteria, where thick septa (>2 mm) increased malignant potential by 4.9-fold (95 % CI 2.3–10.7) [9]. Particularly noteworthy is the ill-defined tumor-muscle interface accompanied by a CT attenuation gradient >50 HU/cm, which corroborates Kransdorf's finding that non-adipose mass-like regions elevated malignant risk by 32-fold (OR = 31.8, 95 % CI 1.6–616.5) [9]. However, it must be emphasized that these imaging features are specific to this particular case. The manifestation of indistinct tumor margins shows considerable variation across different cases and should not be considered a universal diagnostic criterion. Furthermore, in a retrospective analysis of MRI findings from 87 histologically confirmed lipomatous tumor patients, Brisson et al. demonstrated that age > 60 years, tumor diameter > 10 cm, and presence of non-adipose areas increased the likelihood of ALT/WDL by 2.61- to 6.25-fold [10]. Although MRI was not performed in the current case, the study by Brisson et al. established important MRI diagnostic criteria for lipomatous tumors. Their research identified key discriminative features on MRI: (1) non-adipose components (OR = 6.25), (2) lesion size >10 cm (OR = 5.43) and (3) thick/nodular septa (with nodules >1 cm being exclusively observed in ALT/WDL). Notably, the study reported that MRI demonstrated high sensitivity (90.9 %) but limited specificity (37.0 %) for diagnosing ALT/WDLS due to overlapping features with lipomas, thus definitive diagnosis still requires molecular confirmation [10].

In terms of immunohistochemistry and molecular genetics, ALT/WDLS is characterized by the presence of multi-ring or giant chromosomes derived from the long arm of chromosome 12. This cytogenetic abnormality leads to the amplification of the chromosomal region 12q13–15, which results in the amplification of genes such as MDM2, CDK4, HMGA2, and TSPAN31. Among these, the proteins encoded by MDM2 and CDK4 play a role in cell cycle regulation and are considered driving genes for this tumor type [11,12]. Therefore, immunohistochemical detection of overexpression of MDM2 and CDK4 proteins serves as an effective diagnostic tool. In this case, immunohistochemistry showed positive expression of both MDM2 and CDK4. Combined with the patient's medical history and preoperative CT suggesting chest wall invasion, benign lipoma was excluded, and the diagnosis of right breast ALT/WDLS was made. Additionally, research indicates that carboxypeptidase M (CPM), a protein encoded by the CPM gene, serves as a novel tumor marker and aids in the differentiation of ALT/WDLS from benign lipomas and normal adipose tissue. In this study, amplification of CPM was consistently observed in all 32 ALT/WDLS cases (100 % sensitivity), while no amplification was detected in lipomas or normal fat samples (100 % specificity). These findings were further validated by both fluorescent in situ hybridization (FISH) and chromogenic in situ hybridization (CISH), demonstrating the robustness of CPM as a diagnostic tool. Future studies should explore the potential role of CPM in tumor progression and its utility in long-term patient follow-up to assess prognostic significance [13].

## 4 Conclusion

The diagnosis of this breast ALT/WDLS with pectoralis major muscle invasion highlights the clinical challenges of such tumors. Although the patient had no significant family history, typical risk factors were present: middle-aged onset (peak incidence 50–70 years), deep muscle infiltration growth pattern, and characteristic imaging findings of ill-defined borders with surrounding tissue invasion. The definitive diagnosis was achieved through histopathological examination (atypical stromal cells in fibrous septa) combined with molecular testing (MDM2/CPM amplification), emphasizing the need for vigilance regarding the dual clinical features of age > 50 years coupled with infiltrative growth in atypical lipoma-like masses. Although ALT/WDLS is intermediately malignant, its resemblance to benign lipomas often leads to misdiagnosis, making pathological evaluation and molecular testing crucial for differential diagnosis. Surgical resection remains the mainstay of treatment. Postoperative multimodal surveillance is recommended: chest CT every 6 months during the first 2 years to monitor recurrence and distant metastasis, along with annual testing of MDM2/CDK4 and related genes, continuing for at least 5 years. For high-risk patients with positive margins or extensive local invasion, follow-up intervals should be shortened to 3 months. This case not only alerts clinicians to maintain a high index of suspicion for deep-seated lipoma-like masses, but also underscores the need to develop molecular stratification-based individualized follow-up protocols to optimize long-term management of these frequently misdiagnosed tumors.

## Consent

Written informed consent was obtained from the patient for publication of this case report and accompanying images. A copy of the written consent is available for review by the Editor-in-Chief of this journal on reques**t.**

## Ethical approval

This study has obtained ethical approval.

## Sources of funding

This study has been funded.

## Research registration number

Our case-report is not a ‘First in Man’ study, so is not subject for registration in clinical trial websites.

## Conflicts of interest

No potential conflicts of interest were disclosed.

## References

[1] Kerwan A., Al-Jabir A., Mathew G. (2025). Revised surgical CAse REport (SCARE) guideline: an update for the age of artificial intelligence. Premier Journal of Science.

[2] Mankin H.J., Mankin K.P., Harmon D.C. (2014). Liposarcoma: a soft tissue tumor with many presentations. Musculoskelet. Surg..

[3] Dei Tos A.P. (2014). Liposarcomas: diagnostic pitfalls and new insights. Histopathology.

[4] Mahmood U., Nguyen J.D., Chang J. (2009). Atypical lipomatous tumor/well-differentiated liposarcoma of the parotid gland: case report and literature review. Ear Nose Throat J..

[5] Evans H.L., Soule E.H., Winkelmann R.K. (1979). Atypical lipoma, atypical intramuscular lipoma, and well differentiated retroperitoneal liposarcoma: a reappraisal of 30 cases formerly classified as well differentiated liposarcoma. Cancer.

[6] Dei Tos A.P., Pedeutour F., Fletcher C.D., Uni K.K., MertensF (2002). World Health Organization Classification of Tumors: Pathology and Genetics of Tumors of Soft Tissue and Bone.

[7] Salemis N.S. (2015). Intramuscular atypical lipomatous tumor/well-differentiated liposarcoma of the pectoralis major masquerading as a breast tumor: management and review of the literature. Int. Surg..

[8] Javery O., Jagannathan J.P., Saboo S.S. (2012). Case report: atypical lipomatous tumor with unusual extensive metaplastic ossification. Cancer Imaging.

[9] Brisson M., Kashima T., Delaney D. (2013). MRI characteristics of lipoma and atypical lipomatous tumor/well-differentiated liposarcoma: retrospective comparison with histology and MDM2 gene amplification. Skeletal Radiol..

[10] Kransdorf M.J., Bancroft L.W., Peterson J.J. (2002). Imaging of fatty tumors: distinction of lipoma and well-differentiated liposarcoma. Radiology.

[11] Fletcher C.D., Akerman M., Dal Cin P. (1996). Correlation between clinicopathological features and karyotype in lipomatous tumors. A report of 178 cases from the chromosomes and morphology (CHAMP) collaborative study group. Am. J. Pathol..

[12] Laco J., Mentzel T., Hornychova H. (2009). Atypical lipomatous tumors of the tongue: report of six cases. Virchows Arch..

[13] Erickson-Johnson M.R., Seys A.R., Roth C.W. (2009). Carboxypeptidase M: a biomarker for the discrimination of well-differentiated liposarcoma from lipoma. Mod. Pathol..

